# Surveillance of infections in long-term care facilities (LTCFs): The impact of participation during multiple years on health care-associated infection incidence

**DOI:** 10.1017/S0950268819001328

**Published:** 2019-09-09

**Authors:** A. P. J. Haenen, L. P. Verhoef, A. Beckers, E. F. Gijsbers, J. Alblas, A. Huis, M. Hulscher, S. C. de Greeff

**Affiliations:** 1 National Institute for Public Health and the Environment, Centre for Infectious Disease Research, Epidemiology and Surveillance, Bilthoven, The Netherlands; 2 Radboud University Medical Center, Radboud Institute for Health Sciences, IQ healthcare, Nijmegen, The Netherlands; 3 Vivium Careg Group, Long-Term Care Facility Naarderheem, Naarden, The Netherlands

**Keywords:** Incidence, infectious disease epidemiology, surveillance system

## Abstract

We studied trends in the incidence of health care-associated infections (HAIs) in LTCFs between 2009 and 2015 and determined the effect of participation in our network. Elder-care physicians reported weekly the number of cases of influenza-like illness, gastroenteritis, (probable) pneumonia, urinary tract infections (UTIs) and all-cause mortality. Trends in the incidence of infection and mortality in relation to LTCF characteristics were calculated using multilevel univariate and multivariate logistic regression. Thirty LTCF participated for 3 years or more, 16 for 2 years and the remaining 12 LTCF for 1 year. During the study period, the median number of beds decreased from 158 to 139, whereas the percentage of residents with private bedrooms increased from 14% to 87%. UTIs were the most frequently reported infections, followed by (probable) pneumonia and gastroenteritis. Adjusted for calendar year and season, we observed a statistically significant decrease in the incidence of influenza-like illness (odds ratio (OR) = 0.8, *P* < 0.01) and (probable) pneumonia (OR = 0.8, *P* < 0.01) for each extra year an LTCF participated. Although there are other likely contributors, such as more private rooms and enhanced infection control measures, the decreasing trend of HAI in LTCFs participating in surveillance implies that surveillance is a valuable addition to current strategies to optimise infection control.

## Introduction

Elderly people have a higher risk of acquiring an infection and of experiencing a more severe disease course following infection than other age groups have [1–3], as a result of underlying chronic illnesses, functional impairment, malnutrition and polypharmacy. Frail elderly residents in long-term care facilities (LTCFs) are especially at risk of acquiring health care-associated infections (HAIs) due to their dependence on care, sharing of facilities with other residents and living in a confined environment [4–6]. Infections in residents of LTCFs have been associated with high rates of morbidity and mortality and substantial health care costs [4]. HAIs are defined as infections occurring after exposure to health care and often, but not necessarily, as a consequence of this exposure [7]. The most reported HAIs in LTCFs are urinary tract infections (UTIs), respiratory tract infections, skin or tissue infections and gastro-intestinal infections [8, 9].

A number of studies on HAIs in long-term care settings in several European countries have reported incidences of HAIs between 2.7 and 11.8 per 1000 resident days [5, 10–14] and prevalence between 2.2% and 4.4% [15–17]. However, it is difficult to compare the incidences and prevalence reported in these studies, due to differences in methodology (e.g. definition of HAIs and duration of follow-up) and study population/case mix between countries.

Most HAIs are assumed to be preventable, or at least the risk of acquiring an HAI can be reduced by adequate infection prevention measures and timely identification of infected persons may help to stop transmission. In recent years, increasing attention has been given to infection prevention control measures in the LTCF setting [18–21]. In the Netherlands, a national sentinel surveillance network to study infectious diseases and HAIs in LTCFs (SNIV) started in 2009 [22, 23]. The aim of the SNIV network is to collect and analyse year-round surveillance data systematically on the incidence of various infections and overall mortality in Dutch LTCFs to facilitate local interventions and national policymaking on the prevention and control of HAIs in LTCFs. In addition, surveillance data are important to analyse trends over time to see whether any improvement follows feedback and to see whether long-term participation has an added value in preventing infections in Dutch LTCFs.

In this paper, we describe trends in HAI incidence in the Netherlands from 2009 to 2015 and study whether long-term participation of LTCFs in a national surveillance network affects trends in HAI incidence.

## Methods

### Participants and setting

The SNIV network is designed as an ongoing active sentinel surveillance network on infectious diseases and HAIs. Nursing homes were recruited through the academic networks in the Netherlands and by a nationwide mailing to all the nursing homes to get a representative network for the country. All LTCFs in the Netherlands with more than 50 residents can enroll-and withdraw-at any time in the network. LTCFs are approached by mail, leaflets and symposia to participate voluntarily in the network; further details were described previously [23].

### Reported surveillance data

If enrolled, elder-care physicians and/or nurse practitioners are requested to report, through the web-based surveillance system, the weekly aggregated number and age category of new cases of gastroenteritis, influenza-like illness and/or (probable) pneumonia, as well as weekly mortality in their LTCF. From 2011 onwards, the aggregated number of residents with a UTI was also registered within the surveillance network. Infections were based on clinical definitions that are used in common practice in the Netherlands (Table 1). Regarding gastroenteritis, not only new cases were reported; physicians and/or nurse practitioners also reported whether cases were part of an outbreak. An outbreak of gastroenteritis is defined as three or more residents from a single ward or unit, or 3% or more of the entire facility, who developed diarrhoea and/or vomiting within a 7-day period.

**Table 1.** Clinical definitions of health care-associated infections registered in SNIV

Gastroenteritis	The resident must have one of the following four conditions: diarrhoea: three or more episodes in 24 h, deviating from normal person diarrhoea and two of the following symptoms: fever, vomiting, nausea, stomach ache, abdominal cramps, blood or mucus in stool vomiting and two of the following symptoms: fever, nausea, stomach ache, abdominal cramps, blood or mucus in stool vomiting three or more episodes in 24 h (without other symptoms and vomiting is not related to the use of medication)
Influenza-like illness	The resident must meet the following conditions: An acute start of symptoms *And* at least one of the following systemic symptoms: fever or febrile feeling, malaise, headache, myalgia *And* at least one of the following three respiratory symptoms: cough, sore throat, shortness of breath
(Probable) pneumonia	The resident must have at least one of the following symptoms and be suspected of lower respiratory infection, probably pneumonia, as they occur as change compared to the former situation and other likely diagnoses are excluded: Tachypnea, malaise, confusion, shortness of breath, cough (productive or unproductive), fever > 38 °C or fever in the past 48 h, pain in the chest (respiratory) *And* with new focal (unilateral) abnormalities on auscultation of the lungs
UTI	The resident must have (based on the guideline by the Dutch Association of Elderly Care Physicians): General or urinary-related symptoms (painful or, frequent urination, abdominal symptoms, anorexia, increased confusion, drowsiness, fatigue, increased incontinence or urine and reduced mobility, in the absence of a source of infection elsewhere). *And* signs of inflammation (detected by microscopic examination or by leukocyte esterase test or urine sediment) *And* a bacteriuria (determined with nitrite test or urine culture (not applicable to catheter use)).

Annually, the general characteristics of all participating LTCFs are collected through a web-based questionnaire; that is, for each subsequent year a LTCF participates in the network, a new questionnaire is filled out. These characteristics include number of beds per facility, size of the wards, number of private/shared rooms and bathrooms, amount of staff exchange between wards (%), influenza vaccination (%) uptake among residents and among health care workers and the presence of an infection control committee. The amount of staff exchange between wards is collected to be able to answer our hypothesis that it might be that interchange of health care workers facilitates the spread of pathogens that cause gastroenteritis and whether there are problems with hand hygiene.

### Data analysis

Descriptive statistics were used to describe the general characteristics of LTCFs through the various years.

Weekly incidence of gastroenteritis, influenza-like illness, (probable) pneumonia, UTI and overall mortality were calculated per 1000 resident-beds by dividing the total number of new cases in 1 week by the total number of residents in all the participating LTCFs in that week (resident weeks) × 1000. For incidence calculations, the total number of residents was based on the yearly reported number of beds, assuming 100% occupancy. To determine whether participation in a HAI surveillance network during several years affected HAI incidence, the GLIMMIX procedure in SAS for Windows was used for multilevel univariate and multivariate logistic regression by comparing each surveillance year with the previous year while correcting for the calendar year and seasonal trends. A random intercept was included for LTCFs as a correction factor for a possible cluster effect. The outcome variable was infection yes/no at the patient level for each of the investigated infectious diseases separately. Factors analysed were: onset month, onset season, onset calendar year and duration of participation, with the latter kept fixed in the model because this was our factor of interest. For the other factors, the strongest predictors were chosen while using a minimum of the degrees of freedom. If statistically significant (*P* = 0.05), predictors remained in a multivariate model. The final model was assessed for the goodness of fit.

Descriptive statistics were also used to study differences in the size and mean duration of outbreaks accounting for the number of years participating in the surveillance study. An outbreak included the number of cases reported in successive weeks, with the first outbreak-free week indicating the end of the outbreak. Duration of outbreaks was measured in weeks, size of outbreaks in a total number of incident cases of gastroenteritis. Statistical differences were calculated using the *t*-statistic, using the proc means procedure in SAS. An outbreak of gastroenteritis was defined as three or more residents from a single ward or unit, or 3% or more of the entire facility, who developed diarrhoea and/or vomiting within a 7-day period. The number of cases per outbreak, as reported per week, was summarised for all successive outbreak-weeks.

Statistical analyses were performed in SAS for Windows, version 9.3 (SAS Institute Inc., USA).

## Results

### General characteristics of LTCFs

**Fig. 1.**
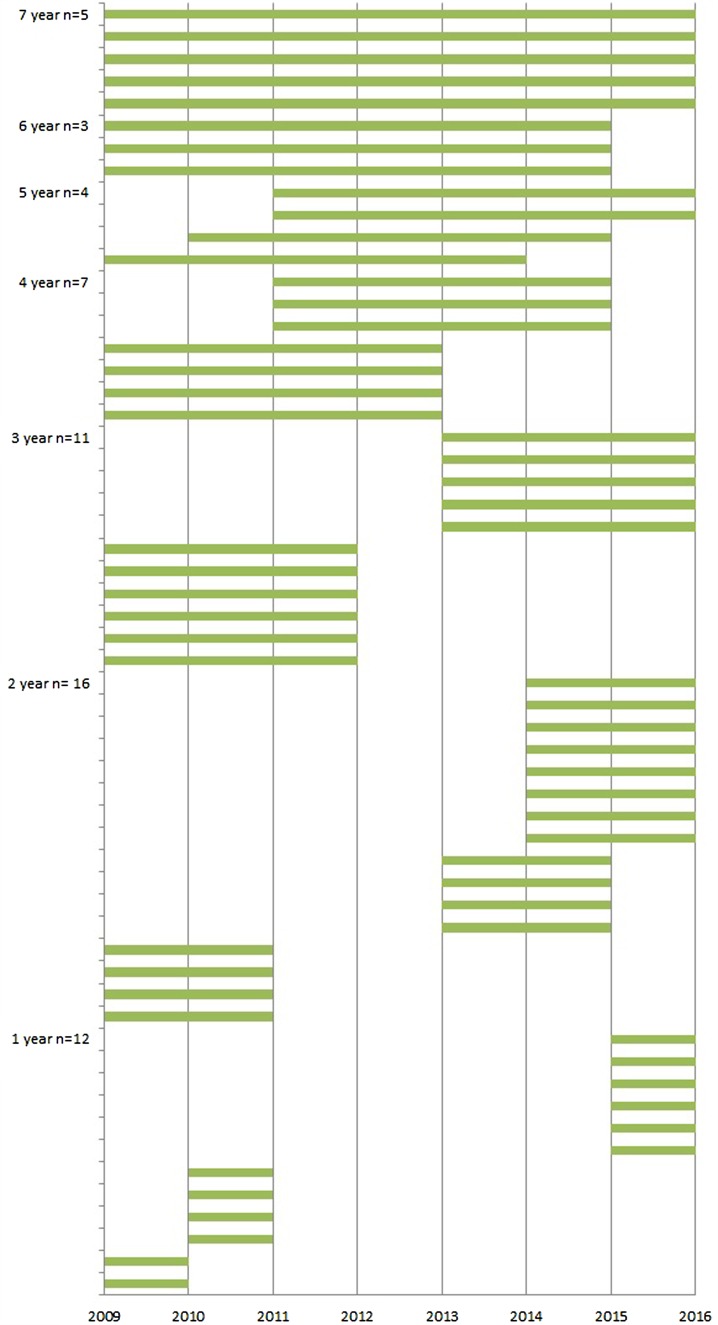
Years of participation in the SNIV network per LTCF.

The average number of participating LTCFs was 25 (range 18–25) each year from 2009 through 2015. In total, 58 LTCFs participated in the surveillance network in this period; five of these LTCFs participated for 7 years, three for 6 years, four for 5 years, seven for 4 years, 11 for 3 years, 16 for 2 years and the remaining 12 for 1 year (Fig. 1). Since the start of the surveillance network, the median number of beds per LTCF decreased from 158 to 139 and, as a result, the number of resident weeks the LTCFs registered infections also decreased. The exception was 2014; in that year, more facilities participated, so the number of resident weeks increased although the number of residents per LTCF was the lowest of all years. The percentage of residents with private bedrooms increased from 14% to 87% and at the same time, the percentage of residents with a private bathroom increased in the first 3 years and stabilised in 2012 to around 30%. The amount of staff exchange between wards initially decreased but, from 2012 onwards, increased again to 51%, to as high as in 2009 (Table 2).

**Table 2.** General characteristics of the SNIV-participating LTCFs by year

Institutional characteristics	2009	2010	2011	2012	2013	2014	2015
LTCFs participating (*N*)	25	28	25	19	24	30	23
Resident weeks	177 677	158 628	136 746	109 452	121 377	157 875	143 503
Residents (total)	3417	3050	2630	2104	2334	3036	2708
Bed capacity (median, range)	158 (62–284)	130 (41–234)	128 (56–234)	124 (56–199)	113 (16–406)	99 (16–417)	139 (13–230)
Number of health care workers (median, range)	270 (70–680)	292 (20–619)	199 (64–451)	189 (64–529)	122 (40–597)^a^	91 (19–200)^a^^/^^b^	83 (23–282)^a^^/^^b^
Private bedrooms (⩾50% of residents haves private bedroom)	14%	49%	59%	76%	90%	90%	87%
Private bathrooms (⩾50% of residents haves private bathroom)	0%	9%	24%	33%	30%	29%	30%
LTCFs with infection control committee (%)	88%	71%	72%	95%	96%	100%	95%
Staff exchange between wards (%)	51%	33%	16%	31%	49%	41%	52%
Influenza vaccination coverage: residents (median, range)	92% (70%–99%)	95% (70%–98%)	95% (70%–100%)	95% (70%–100%)	94% (67%–99%)	90% (67%–100%)	95% (75%–100%)
Influenza vaccination coverage: health care workers (median, range)	16% (4%–52%)	20% (5%–50%)	19% (4%–65%)	17% (5%–50%)	15% (5%–30%)	15 (7%–60%)	15% (0%–60%)
Shared living space (median, range) (number of rooms where residents share the environment)	5 (1–13)	5 (1–18)	5 (1–20)	5 (1–20)	5 (1–20)	5 (1–20)	5 (2–22)

a The number of healthcare workers is an optional variable in the 2013, 2014 and 2015 questionnaire. Seven LTCFs (29%) provided data regarding health care workers in 2013–2014; 18 LTCFs (78%) in 2015.

b In 2014, the number of health care workers was provided per caring unit and these numbers were summarised into a total. This number may not be comparable to previous years due to the different methods used.

### Trend in HAI incidence from 2009 until 2015

The incidence of influenza-like illness, (probable) pneumonia, gastroenteritis and UTIs fluctuated by year (Table 3).

**Table 3.** Overall incidence per infection by year

	Incidence^a^ (95% CI)						
Types of infections/years	2009	2010	2011	2012	2013	2014	2015
Gastroenteritis	3.8 (3.7–4.3)	4.6 (4.5–5.2)	3.7 (3.4–4.1)	2.5 (2.2–2.8)	1.9 (1.7–2.2)	3.0 (2.7–3.3)	2.2 (2.0–2.5)
Influenza-like Illness	1.6 (1.5–1.9)	0.4 (0.3–0.6)	0.5 (0.4–0.6)	1.8 (1.6–2.1)	0.8 (0.7–1.0)	0.6 (0.5–0.8)	0.9 (0.8–1.1)
(Probable) pneumonia	3.6 (3.4–4.0)	3.7 (3.6–4.2)	2.8 (2.5–3.2)	3.5 (3.2–3.9)	3.7 (3.4–4.1)	4.6 (4.2–4.9)	3.8 (3.5–4.2)
UTIs	–	–	8.0 (7.4–8.7)	9.6 (9.0–10.3)	9.5 (8.9–10.0)	11.2 (10.7–11.8)	10.3 (9.8–10.8)

a Incidences per 1000 resident weeks.

**Fig. 2.**
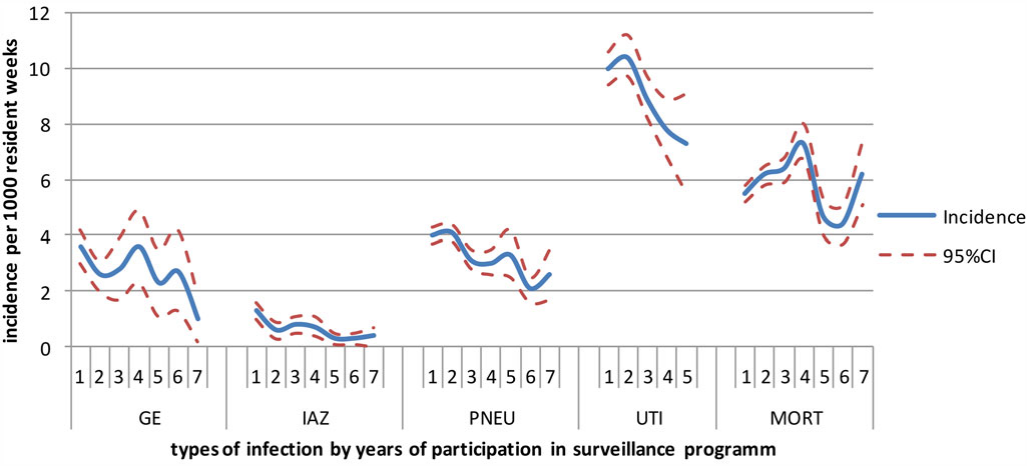
Incidence per 1000 residents weeks per each successive year participating in surveillance. GE, gastroenteritis; IAZ, influenza-like illness; PNEU, probable pneumonia; UTI, urinary tract infections; MORT, mortality.

Figure 2 shows the incidence of influenza-like illness, (probable) pneumonia, gastroenteritis, UTIs and all-cause mortality by the duration of participation in years. UTIs were the most frequently reported infections in the Dutch LTCFs, followed by (probable) pneumonia and gastroenteritis. Table 4 shows the crude, calendar year and season-corrected odds ratios for incidence in each subsequent year during surveillance. After correction for the calendar year and season, the incidence of influenza-like illness and (probable) pneumonia decreased over time (OR: influenza-like illness = 0.8, *P* < 0.01; and OR: lower respiratory tract infections = 0.8, *P* < 0.01); the risk of contracting a lower respiratory tract-infection decreased by 20% in LTCFs participating each year in the surveillance network.

**Table 4.** Results of the multilevel logistic regression analysis to find the effect of participation duration on incident cases (yes, no) of different infectious diseases in 58 LTCFs

Crude OR (95% CI)		OR (96% CI) Adjusted for calendar year and season	*P*-value
Gastroenteritis	0.9 (0.85–0.91)	1.0 (0.85–1.07)	0.40
Influenza-like illness	0.7 (0.70–0.79)	0.8 (0.67–0.91)	<0.01
(Probable) pneumonia	0.9 (0.87–0.92)	0.8 (0.76–0.91)	<0.01
UTI	1.0 (0.93–0.98)	1.0 (0.88–1.15)	0.96
Mortality	1.0 (0.94–0.97)	1.0 (0.95–1.08)	0.73

The following variables were analysed: incidence yes/no (outcome variable), onset week (1–52), onset month (1–12), onset season (1–4, corresponding to spring, summer, autumn, winter), onset years (1–7, corresponding to 2009–2015), duration of participation (1–7). Duration of participation was kept fixed in the model. The strongest predictors remained in the final model: season and calendar year.

### Gastroenteritis outbreaks

A total number of 123 gastroenteritis outbreaks were reported between 2009 and 2015 in the 58 LTCFs that participated in the network during this period. The mean duration of outbreaks was 1.7 (95% CI 1.6–1.9) weeks and on average 13 (95% CI 11–15) cases were involved per outbreak. No significant differences in outbreak characteristics such as size and mean duration were seen after prolonged participation (Table 5).

**Table 5.** Number, size and duration of outbreaks of gastroenteritis, by duration of participation in the surveillance network

Number of years participating in the surveillance network^a^	Number of LTCFs with outbreaks of total (%)	Number of outbreaks	Mean duration of outbreaks in weeks (95% CI)	Mean number of cases per outbreak (95% CI)
1	18/58 (31)	32	1.8 (1.5–2.2)	11.7 (6.7–16.6)
2	19/46 (41)	33	1.8 (1.4–2.2)	13.3 (8.5–18.2)
3	15/30 (50)	23	1.3 (1.1–1.5)	13.7 (7.3–20.0)
4	6/19 (32)	11	2.2 (1.5–2.9)	14.5 (8.7–20.2)
5	7/12 (58)	12	1.6 (1.0–2.2)	11.3 (6.0–16.5)
6	2/8 (25)	4	1.8 (0.2–3.3)	23.8 (0–52.1)
7	1/5 (20)	3	1 (–)	5.0 (2.5–7.5)

a Five LTCFs participated for 7 years, three for 6 years, four for 5 years, seven for 4 years, 11 for 3 years, 16 for 2 years and the remaining 12 for 1 year.

## Discussion

Although the incidence of infections fluctuated per year, the incidence of influenza-like illness and probable pneumonia significantly decreased over time for each extra year a LTCF participated in the SNIV network. This decline was irrespective of the calendar year or season at the start of participation. We also observed a decrease in the incidence of UTIs and gastroenteritis after long-term participation in the surveillance; however, this decrease was not statistically significant. Despite not knowing whether, and to what extent, the LTCFs have performed or applied interventions in recent years, the decrease of infections, although not all significant, suggest a possible positive effect of (prolonged) participation in an infectious disease surveillance programme in LTCFs, as Geubbels *et al*. also saw in hospitals [24].

Changes in the organisation and infrastructure over time may affect the level of infection prevention and control measures and therefore affect the incidence of HAIs. We observed general changes such as a reduction in number of beds and an increased number of private bedrooms. Wendt *et al*. found that staying in an LTCF in a room with three or more beds was positively associated with the transmission of *Staphylococcus aureus* [25]. Despite that in a multicentre hospital study the rates of adherence to hand hygiene were better in private rooms, there is no stringent evidence linking LTCF design and construction with improving the incidence of infections [26]. It is therefore unlikely that the decrease in infections is related to structural LTCF changes.

Although the incidence of respiratory infections decreased significantly, we did not find a significant decrease in the incidence of gastroenteritis and UTIs. This may be related to the availability of private rooms and bathrooms. Despite having their own bedroom, most residents still share a bathroom in LTCFs. A shared bathroom can be a reservoir of microorganisms, which can spread to other residents directly by the hands of health care workers or indirectly from materials and medical supplies. The Dutch health inspectorate from the Ministry of Health, Welfare and Sport concluded in April 2015 that LTCFs still do not take sufficient action to improve infection prevention regarding, for example, the cleaning of surfaces and bedpans. Wolf *et al*. found the transmission of bacteria from the sink to patients [27]. Transmission of microorganisms could happen even more often when clean and dirty actions take place in the same room, at the same sink. In relation to this, private bathrooms for all the clients or at least a well-placed, equipped sink for performing hand hygiene for employers is needed [28].

We have not included other (nationwide) developments in LTCFs that may have affected the incidence of HAIs. Although some changes have been made in past years in the admission policy with respect to resident conditions [29], we have no indication that the population in the SNIV LTCFs has become more or less vulnerable to infectious diseases.

The effect of prolonged participation is likely affected by a mixture of patient and LTCF characteristics, which is difficult to disentangle, due to the aggregated nature of reporting of the described surveillance data in this study. Although this choice for aggregated data at the LTCF level enabled us to keep the surveillance system user-friendly with a relatively low workload, it can also be seen as a limitation of our study because we cannot establish direct relationships with risk factors at the client level. To identify client-related risk factors that could help develop best-practice measures to protect frail elderly against HAIs, information at the client level is required for clients with and without an infection, which would substantially increase the registration workload. This will, however, change when registration of infections is embedded in the electronic patient records, for which developments are underway in many LTCF in the Netherlands.

Participation in the network is voluntary and LTCFs have the opportunity to start and stop the registration whenever they want. There are several reasons LTCFs no longer participate in the network. They may find it too time-consuming or the elderly care physician who performs the registration leaves the LTCF and nobody takes over. We hope the use of electronic patient records will render participation less time-consuming so that more LTCFs will participate for a longer period of time.

Even though we do not know whether and how the LTCFs use the feedback from the surveillance data [16], there are other likely contributors such as more private rooms and enhanced infection control measures, our study demonstrates that surveillance is a valuable addition for LTCFs to adopt in their infection control strategies. Participation in surveillance probably helps increase awareness and facilitates the implementation of (targeted) infection prevention and control practices.

Given the increasing worldwide threat of antimicrobial resistance, improvement of infection prevention and control practices is also important to prevent the spread of resistant microorganisms and to prevent infections in LTCF residents.

To improve the value and impact of our surveillance network further, we are planning to include microbiological diagnostics for case definitions and antibiotic usage data in the surveillance. This, and an already started in-depth study to improve hand hygiene compliance [30] will provide the SNIV network with even more information for action to control HAIs in LTCFs.
